# The potential mechanism of Saikosaponin D against luminal A breast cancer based on bioinformatical analysis, molecular docking and in vitro studies

**DOI:** 10.1186/s41065-025-00510-8

**Published:** 2025-07-24

**Authors:** Lichang Yang, Ru Chang, Jianzhen Pan, Shan Huang

**Affiliations:** 1 Anhui University of Chinese Medicine, Hefei, 230012 China; 2 Nanjing University of Chinese Medicine, Nanjing, 210023 Jiangsu China; 3 Department of Oncology, The First Affiliated Hospital of Harbin Medical University, Harbin, 150001 China; 4 Department of Intensive Care Medicine, Hunan Cancer Hospital, The Affiliated Cancer Hospital of Xiangya School of Medicine Central South University, Changsha, 410003 China; 5 Chongqing Medical and Pharmaceutical College, No.82, Daxuecheng Middle Road, Shapingba District, Chongqing, 401331 China

**Keywords:** Estrogen receptor, Breast cancer, Saikosaponin D, Proliferation, Cell cycle

## Abstract

**Background:**

Saikosaponin D (SSD) has been shown to have the strongest anti-tumor activity. This study aimed to explore the effects and potential molecular mechanism of Saikosaponin D (SSD) against estrogen receptor-positive breast cancer.

**Methods:**

MCF-7 and T-47D cell lines were treated with a series of concentrations of SSD. Growth, cell cycle distribution, and apoptosis tests were performed. Next, potential targets of SSD against breast cancer were predicted. The targets for SSD were collected from HERB database and PharmMapper Server and displayed accoding to degree.

**Results:**

there was a dose-dependent decrease in MCF-7 and T-47D cancer cell viability and the the half maximal inhibitory concentrations were 7.31 ± 0.63 µM and 9.06 ± 0.45 µM, respectively. Treatment with SSD decreased cell proliferation, arrested cell cycle at G1, and induced cell apoptosis. There were 227 potential targets of SSD against breast cancer, among which ESR1 was a hub gene. SSD treatment can reduce the protein levels of estrogen receptor α (ERα), Cyclin D1 (CCND1), and the proto-oncogene c-Myc (c-Myc).

**Conclusion:**

SSD may have therapeutic potential in estrogen receptor-positive breast cancer, may through its suppression on ESR1.

**Supplementary Information:**

The online version contains supplementary material available at 10.1186/s41065-025-00510-8.

## Background

Breast cancer is one of the most prevalent malignant tumors in women. Estrogen receptor-positive (ER^+^) breast cancer accounts for about 75% of all cases of breast cancer [1]. Breast cancer incidence rates are increasing in many countries and regions, according to recent studies of the global burden of disease [2]. Breast cancer incidence is globally varied in line with human development and is identified as the main malignant tumor for women in high-income countries [3]. In addition, the disease burden of breast cancer is increasing rapidly in many Asian countries, particularly those undergoing rapid economic development. Global age-standardized breast cancer incidence is estimated at 48 per 100,000 women [3, 4]. The rate of increase in breast cancer incidence has accelerated in recent years among Chinese women, according to global breast cancer data [5, 6]. An estimated 700,000 women will die from breast cancer in 2020, accounting for 1 in 6 cancer deaths in women [4]. Conventional breast cancer conventional standard-of-care and treatment modalities remains firmly rooted in multimodal therapy (surgery, RT, systemic agents), but is dynamically evolving towards greater personalization based on molecular drivers, risk stratification, and response assessment [7]. The integration of immunotherapy, CDK4/6 inhibitors, potent ADC, and refined biomarker selection defines the current standard, with ongoing research focused on further optimization and overcoming resistance [8]. Therefore, breast cancer has become a truly global health challenge, with considerable unmet medical needs guided by molecular subtyping and biomarker profiling.

Radix Bupleuri, one member of the Bupleurum species, is widely used clinically to treat liver disease, fever, chills, and tumors. With the growing attention to the main active ingredients, the development and utilization of Radix Bupleuri resources has been rapidly on the increase [9]. Saikosaponins, including Saikosaponin D, are the main pharmacochemical constituents of the Bupleurum species [10]. They have multiple pharmacological activities [11]. Through various mechanisms of action, saikosaponins have been shown to play an important role in the treatment of a wide range of cancers [11]. Saikosaponin D (SSD) has been shown to have the strongest anti-tumour activity among saikosaponins [12]. Through multiple mechanisms, SSD exerts anti-tumour effects on a variety of cancer cells [13]. For instance, it can inhibit lung cancer cell proliferation and induce apoptosis/pyroptosis by activating the NF-κB/NLRP3/caspase-1/GSDMD pathway or inhibiting the STAT3 pathway [14, 15]. SSD can inhibit pancreatic cancer via AKT/mTOR, EGFR/PI3K/Akt, or MKK4-JNK pathway [16–18]. For example, SSD can suppress triple-negative breast cancer through targeting β-catenin signaling or inducing autophagy‑independent apoptosis [19, 20]. Therefore, SSD may be important for researching and developing new anticarcinogens. Unfortunately, information regarding the efficacy and potential mechanism of SSD against luminal A breast cancer is lacking.

Therefore, this study was to determine the potential therapeutic effects of SSD in ER^+^ breast cancer. The potential targets of SSD against ER^+^ breast cancer were explored using bioinformatical analysis, and the screened important target was further verified by molecular modeling and in vitro studies.

## Materials and methods

### Cell culture

Two human estrogen receptor-positive luminal A breast cancer cell lines, MCF-7 and T-47D, were purchased from Cell Center of Medical Research Institute (Shanghai, China). MCF-7 cell line was maintained in MEM (containing NEAA)+0.01 mg/ml insulin+10% fetal bovine serum (FBS)+1% Penicillin-Streptomycin Solution (P/S), while T-47D cell line was in DMEM + 10% FBS+1% P/S. All cells were routinely incubated in a 37℃ humidified incubator, in an atmosphere of 5% CO_2_-95% air. SSD was supplied by National Institutes for Food and Drug Control (Beijing, China). ESR1 pcDNA and negative conrol (NC) were purchased from IBSBIO (Shanghai, China).

### Cell viability assay

The cell viability was measured using Cell Counting Kit-8 (Beyotime Biotechnology, China).

Half maximal inhibitory concentration (IC50) determination: Briefly, 100 µl of cell suspension containing 5000 cells were added to wells of 96-well plate. After 24 h, a wide range of concentrations (1, 2, 5, 10, 20, 50, and 100 µM) of SSD were added to the cells. Cells in Sham group were treated with medium containing dimethyl sulfoxide. After 48 h, Cell Counting Kit-8 reagent was added to the wells and cultured for two hours. The optical density (OD) values were measured at 450 nm.

Cell proliferation assay: Briefly, 100 µl of cell suspension containing 2000 cells were added to wells of a 96-well plate. After 24 h, 7 µM of SSD was added to MCF-7 cell culture, while 9 µM SSD was added to T-47D cell culture. Next, the cell plate was cultured for 24, 48, and 72 h. Then, Cell Counting Kit-8 reagent was added for a two-hour culture. The OD values were measured at 450 nm.

### Cell cycle analysis

Cell cycle distributions were analyzed by propidium iodide staining and flow cytometry. MCF-7 or T-47D cells were treated with 7 µM or 9 µM of SSD for 48 h. After being washed, cells were digested with trypsin and centrifuged at 800×g for 3 min. After fixation in 70% ethanol at 4 °C for an overnight period, the cells were stained with a 20 µg/mL propidium iodide-PBS solution containing RNase A for half an hour at 37 °C. The distribution of the cell cycle in different phases was detected on an Aurora CS (Cytek Biosciences, USA) flow cytometer equipped with the Spectroflo software.

### Apoptosis analysis

Apoptosis analysis was performed using the Annexin V-FITC Reagent (Elabscience Biotechnology, China). MCF-7 or T-47D cells were treated with 7 µM or 9 µM of SSD for 48 h. Cells were centrifuged at 300×g for 5 min, then resuspended and counted. 2 × 10^5^ resuspended cells were added 500 µL of diluted 1 × Annexin V Binding Buffer, as well as 5 µL of Annexin V-FITC Reagent and nuclear DNA staining solution. The cell mixture was vortexed and mixed well, and then incubated at room temperature in dark. After the reaction, the cells were immediately tested by flow cytometry.

### Targets prediction

First, breast cancer-related targets were collected using the keyword “breast cancer” from GeneCards (https://www.genecards.org/) and DisGeNET (https://www.disgenet.org/) databases. Target genes for SSD were collected using HERB database and PharmMapper Server. The common targets were obtained using VENN diagram and subjected to STRING platform to obtain the protein-protein interaction among these targets. Then, the interaction network was analyzed and visualized using CytoScape software. Pathway enrichment for these targets was discovered using the Database for Annotation, Visualization, and Integrated Discovery, and the interesting node was highlighted.

### Molecular docking simulations

Preparation of receptor: 3D structure of homology modeling for ESR1 protein (7uj8) was downloaded from RCSB Protein Data Bank database (https://www.rcsb.org/). Then, the structure was processed to delete Chain B and all water and ionized at a pH of 7.5 using PROPKA. The GHECOM algorithm was used to identify potential small molecule binding sites in proteins for use in molecular docking pockets.

Preparation of ligand: Smiles of SSD were imported into Yinfo platform (https://www.yinfotek.com/) to generate a 3D structure. This tool added hydrogen atoms and charges to SSDs and performed energy optimization under the MMFF94 force field.

Molecular docking: The docking was flexible (Vina). The grid center size was X = 20.756, Y = -28.443, and Z = 12.479 and size values were adjusted (X = 26, Y = 26, Z = 26). pH = 7.2.

Molecular dynamics: The first step of molecular dynamics involved solvating the protein-ligand complexes with explicit OPC water molecules. Amber force fields were AMBER ff19SB + BSC1 + OL3 + GLYCAM_06j + lipid17 + GAFF2.

### mRNA expression analysis

The T-47D and MCF-7 cells were treated in the presence or absence of SSD for 24 hours. For ESR1 mRNA expression analysis, total RNA was isolated from cells using High Pure RNA Isolation Kit (Roche, Italy). Total RNA were reverse transcribed into cDNA (Random primers and SuperScript™ II Reverse Transcriptase, Invitrogen, USA). RT-qPCR was performed in 96 wells on an ABI QuantStudio5 (Applied Biosystems, USA). Primer sequences were: 5’-CGGCATTCTACAGGCCAAATTCAG-3’ (forward) and 5’-CTTCTCTTGAAGAAGGCCTTGCAG-3’ (reverse) for ESR1; 5’-GGGAAATCGTGCGTGACATTAAG-3’ (forward) and 5’-TGTGTTGGCGTACAGGTCTTTG-3’ (reverse) for β-actin (ACTB). The determined cycle threshold (Ct) values were normalized to endogenous control (ACTB) using 2^−ΔΔCt^ method.

### Western blot analysis

MCF-7 and T-47D cells (5 × 10^5^) were stimulated with designated doses of SSD. Cellular proteins were extracted using radioimmunoprecipitation assay buffer (Santa Cruz Biotechnology, USA). After centrifugation at a speed of 15,000 × g for 15 min at 4℃, the protein supernatant was extracted and subjected to prepare a protein assay using Bio-Rad Kit. The proteins were heated for 3 min at 85 °C and allowed to separate using SDS-PAGE. Electroblotting was used to transfer the proteins on the gels to an Immobilon polyvinylidene fluoride membrane (Millipore, USA). To block non-specific proteins, 5% skimmed milk was used at the start of the probe. For the detection of each protein of interest, the membranes were then probed with the appropriate primary and secondary antibodies. For ER-α, anti-Estrogen Receptor α (F-10) (1:1000) (Santa Cruz Biotechnology, USA) was used. To probe Cyclin D1 (CCND1) and c-Myc proteins, antibody (1:3000, Cat. #ab134175, Abcam) and c-Myc antibody (Santa Cruz Biotechnology, USA) were used. The secondary antibodies were purchased from Jackson Laboratories (USA). The protein levels were normalized to protein levels of the β-actin.

### Statistics analysis

Data are illustrated as the means ± standard deviation from at least three independent experiments. The log(dose)-response curves from GraphPad software was used to calculate IC50 values.Significance was analyzed using appropriate statistical tests, such as one-way or two-way ANOVA, inferred at *p* ≤ 0.05.

## Results

### IC50s of SSD for MCF-7 and T-47D cells

**Fig. 1:**
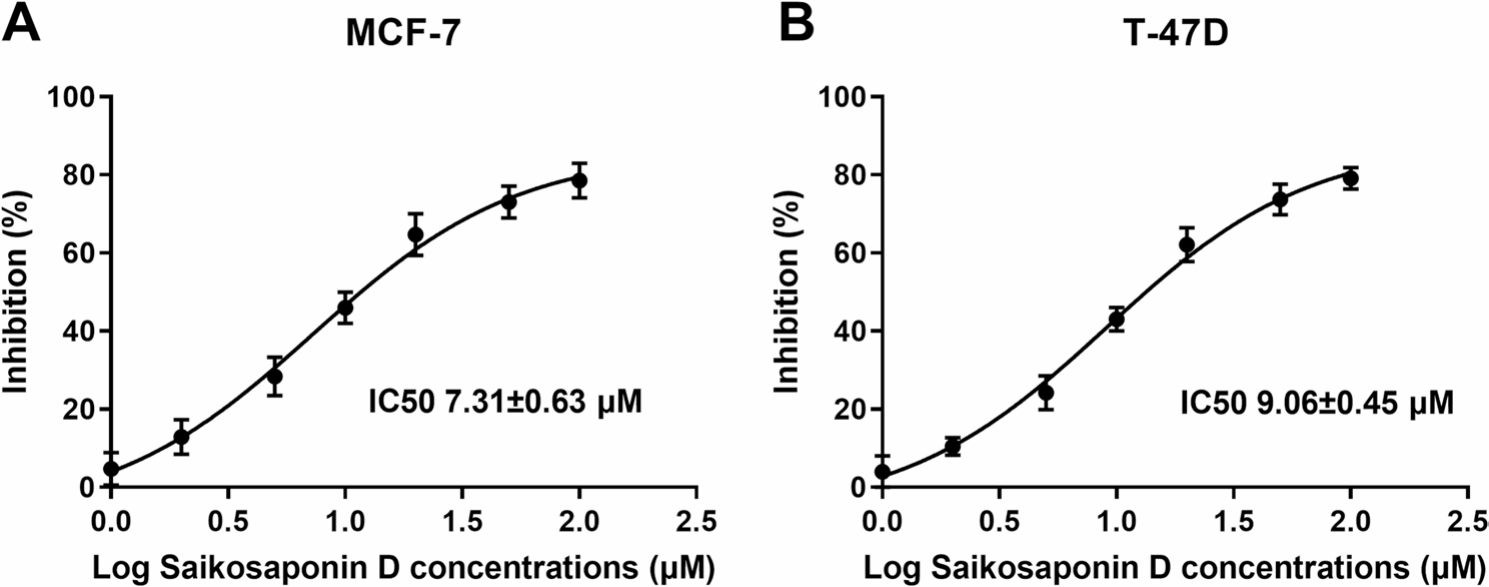
IC50 values of Saikosaponin D toward MCF-7 (**A**) and T-47D (**B**) cell lines

Because of the direct impact it has on the treatment of patients with cancer, accurately predicting an individual’s response to cancer drugs is essential for precision medicine. Over the past decade, a number of large-scale drug screening studies have been carried out using panels of cultured human cancer cell lines. The IC50 is a key parameter [21]. In this study, there was a dose-dependent increase in the inhibition percentage of cell viability of MCF-7 and T-47D cancer cells, and the IC50s were 7.31 ± 0.63 µM and 9.06 ± 0.45 µM, respectively (Fig. 1A and B). Then, 7 µM SSD and 9 µM SSD were used for the MCF-7 and T-47D cell experiments, respectively. 9 µM SSD had no significant effect on MCF-10 A (Figure S1A).

### SSD inhibited MCF-7 and T-47D cell proliferation and induced apoptosis

**Fig. 2:**
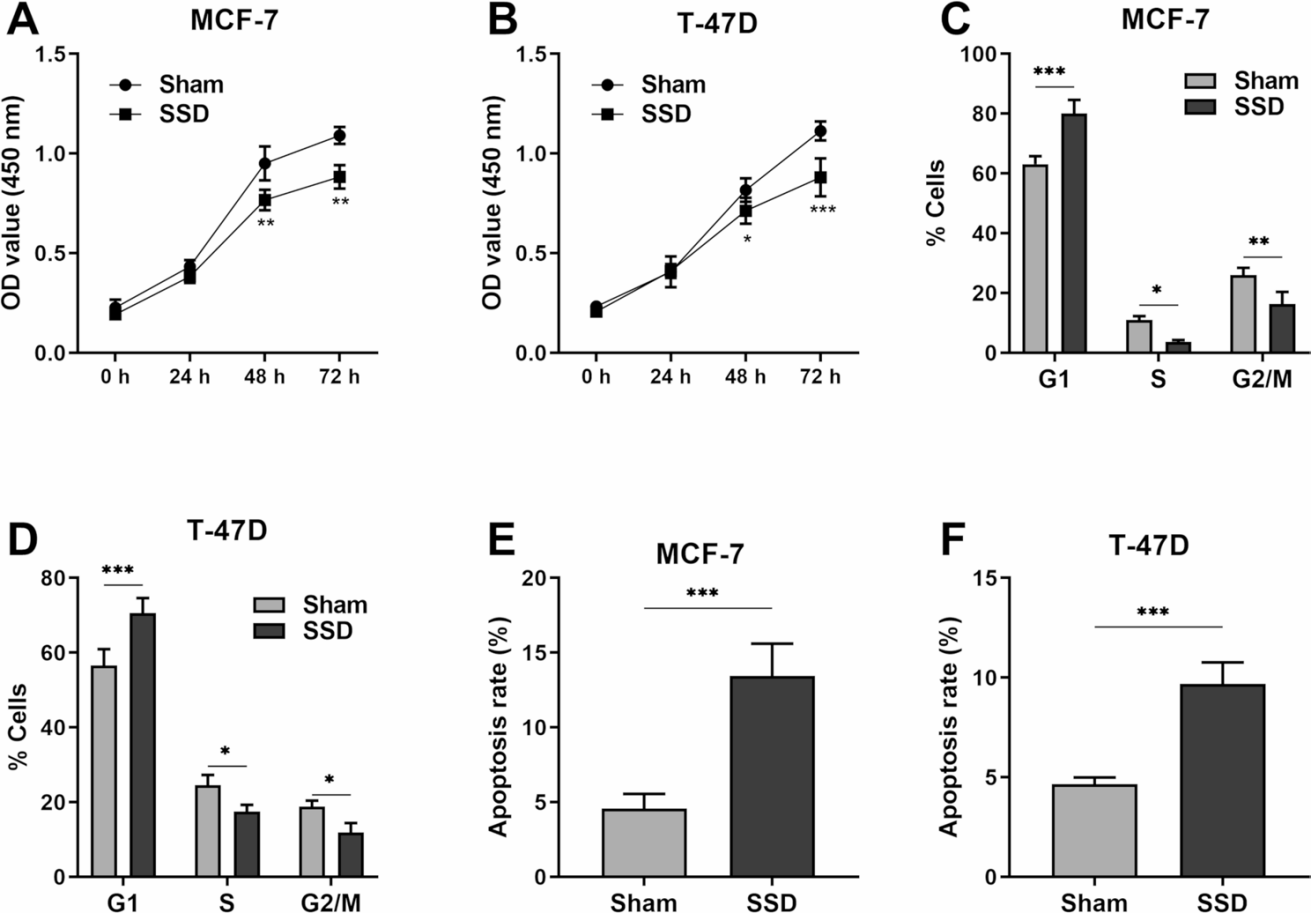
Saikosaponin D inhibited breast cancer cell growth. (**A**) (**B**) Bar graphs indicate the OD values from Cell Counting Kit-8 assays after different days of culture, as indicated. *p < 0.05, **p < 0.01, ***p < 0.001 (Two-way ANOVA with the Bonferroni post hoc test). (**C**) (**D**) Bar graph representing the percentage of cells in G1, S, and G2/M cell cycle phases under Saikosaponin D treatment. *p < 0.05, **p < 0.01, ***p < 0.001 (Two-way ANOVA with the Bonferroni post hoc test). (**E**) (**F**) Bar graphs indicate the percentage of apoptosic cells. ***p < 0.001 (Unpaired t test)

Anticancer drugs can lead to a reduction in tumor size, mainly through inhibition of proliferation and induction of apoptosis of malignant cells. Cell cycle aberrations and apoptosis dysregulation occur during oncogenic transformation. An attractive approach to cancer treatment is to target cell proliferation, cell cycle, and apoptosis. In this study, the anticancer activity of SSD was assessed by proliferation, cell cycle distribution, and apoptosis of MCF-7 and T-47D cells in vitro. As shown in Fig. 2A and B, the proliferation of MCF-7 and T-47D cancer cells was decreased under SSD treatment (*p* < 0.01). To gain a better understanding of the effect of SSD on cell proliferation, we tested whether SSD treatment arrested cells in a specific cell cycle phase. Flow cytometry analysis for different cell cycle phases revealed that cells treated with SSD showed a significant increase in the G1 phase and significant decreases in the S- and G2/M-phase as compared to the sham cells (*p* < 0.05; Fig. 2C and D). Furthermore, apoptosis of MCF-7 and T-47D cancer cells was studied using SSD. It showed SSD at 7 µM induced MCF-7 apoptosis (*p* < 0.001; Fig. 2E), while SSD at 9 µM induced T-47D apoptosis (*p* < 0.001; Fig. 2F). Therefore, SSD can affect the proliferation, cell cycle distribution, and apoptosis of luminal A breast cancer cells.

### ESR1 may be a target of SSD against luminal A breast cancer

**Fig. 3:**
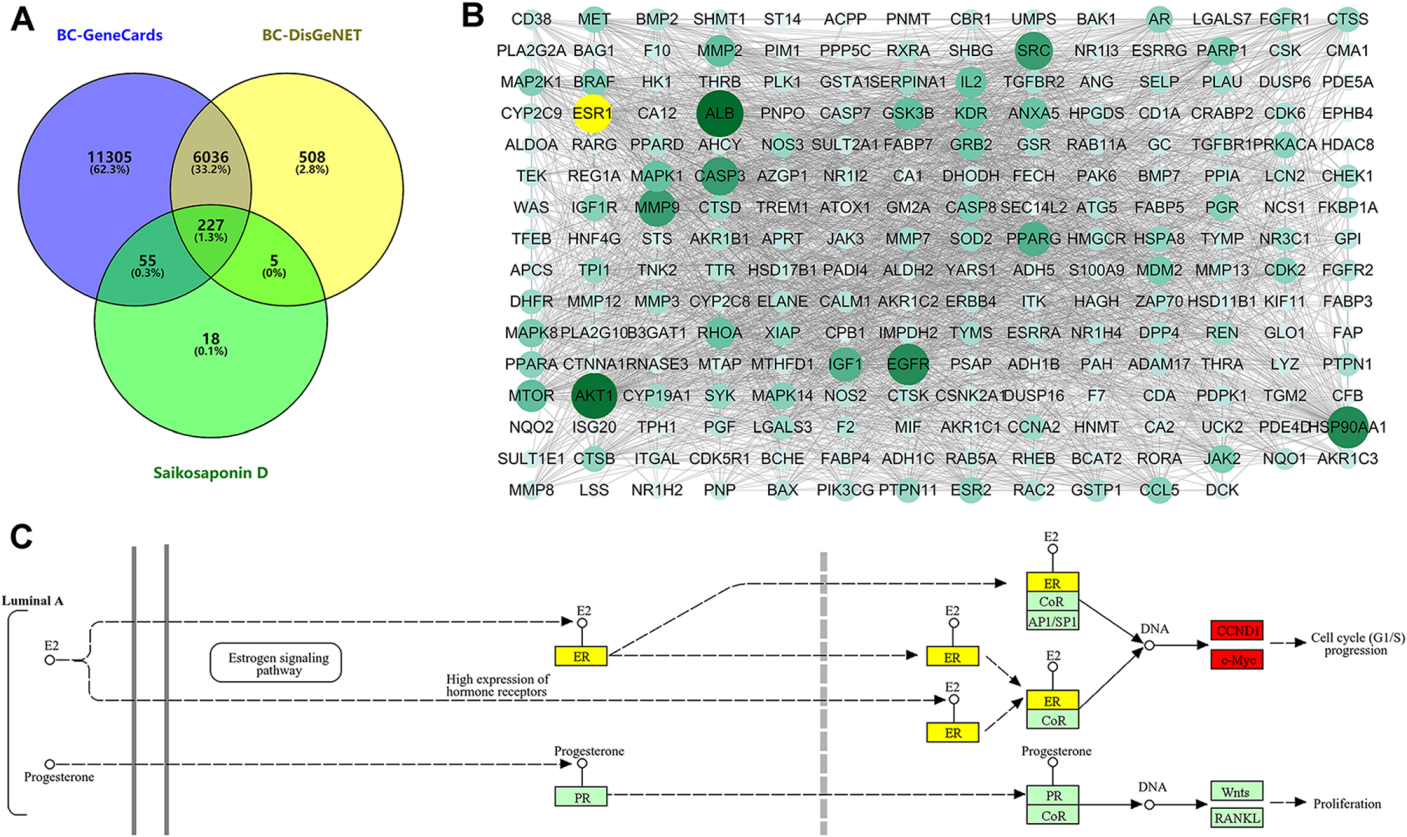
ESR1 may be target of Saikosaponin D against breast cancer. (**A**) The VENN diagram indicnated the overlapped factors between Saikosaponin D targets and breast cancer-related genes (BC-GeneCards and BC-DisGeNET). (**B**) Protein-protein interaction network of the 227 common targets, with size of nodes dependent on degree. (**C**) ESR1 was involved in Liminal A breast cancer pathway

It has been known that there are multiple targets for the antitumor effects of SSD [13]. In this study, we explored the potential targets of SSD against breast cancer. The intersection of SSD-targeting genes and genes of breast cancer resulted in 227 genes (Fig. 3A). The constructed protein-protein interaction network and network analysis results showed that ALB, AKT1, HSP90AA1, EGFR, ESR1, CASP3, MMP9, and SRC were the common hub genes based on “degree”, “MCC”, “MNC” and “closeness” analysis method (Fig. 3B). Then, functional annotation of the 227 genes showed breast cancer pathway was enriched (Figure S1B), and the Luminal A subtype breast cancer involved ESR1 aroused our interest (Fig. 3C).

### Molecular docking between SSD and ESR1

**Fig. 4:**
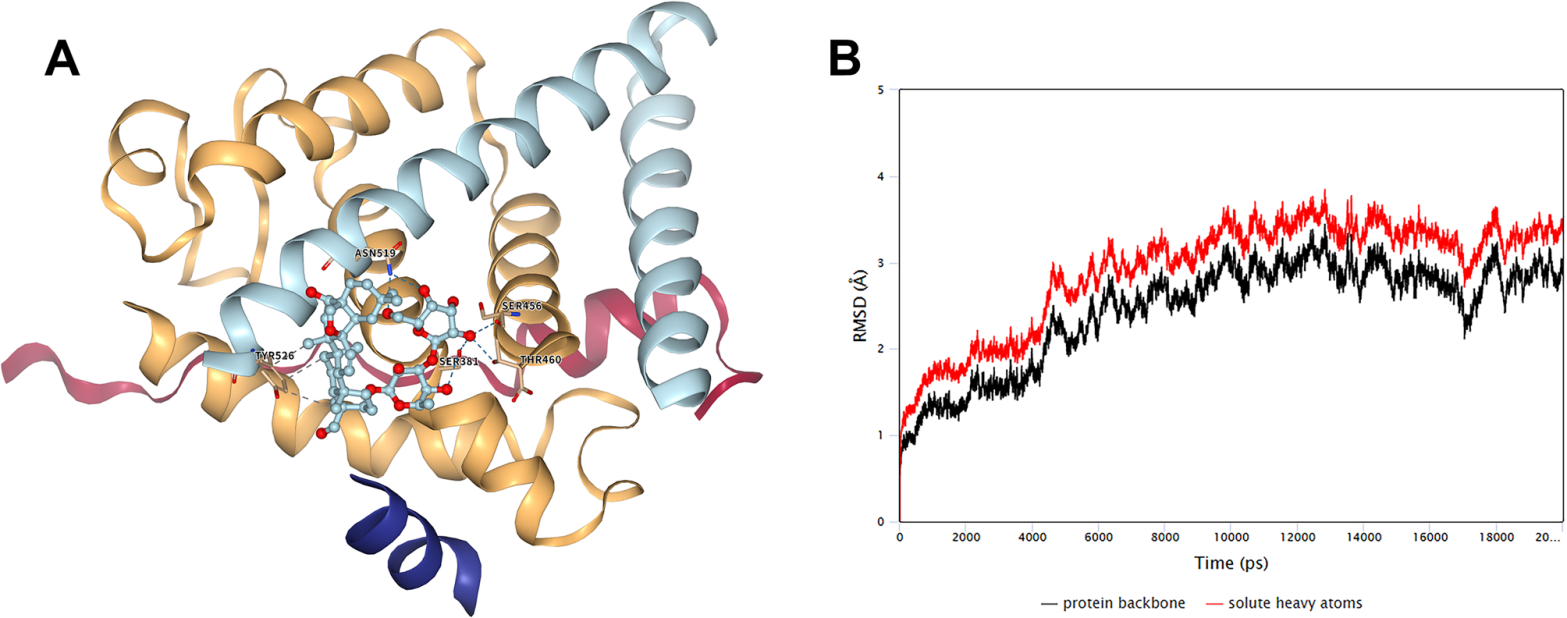
Molecular Docking docking between Saikosaponin D and ESR1. (**A**) The docked models of ESR1 interacting with Saikosaponin D. (**B**) The RMSD value through the molecular dynamics times

To confirm the participation of ESR1 in the SSD mechanism against Luminal A subtype breast cancer, nine poses were identified via Vina methods (Table 1). The best docking pose was shown in Fig. 4A. After molecular dynamics anylsis, the stability of the SSD-ESR1 composite system was well (Fig. 4B).

**Table 1:** The Vina Docking results of Saikosaponin D with ESR1

Pose	Affinity (kcal/mol)	RMSD (lb.)	RMSD (ub.)
1	-6.8	0	0
2	-6.5	3.12	4.97
3	-6.4	3.87	6.28
4	-6.4	6.21	10.68
5	-6.2	3.28	7.22
6	-6.2	4.25	7
7	-6.1	3.67	7.95
8	-6	3.92	7.15
9	-5.9	3.53	8.53

### SSD targeted to ESR1 and regulated CCND1/c-Myc

**Fig. 5:**
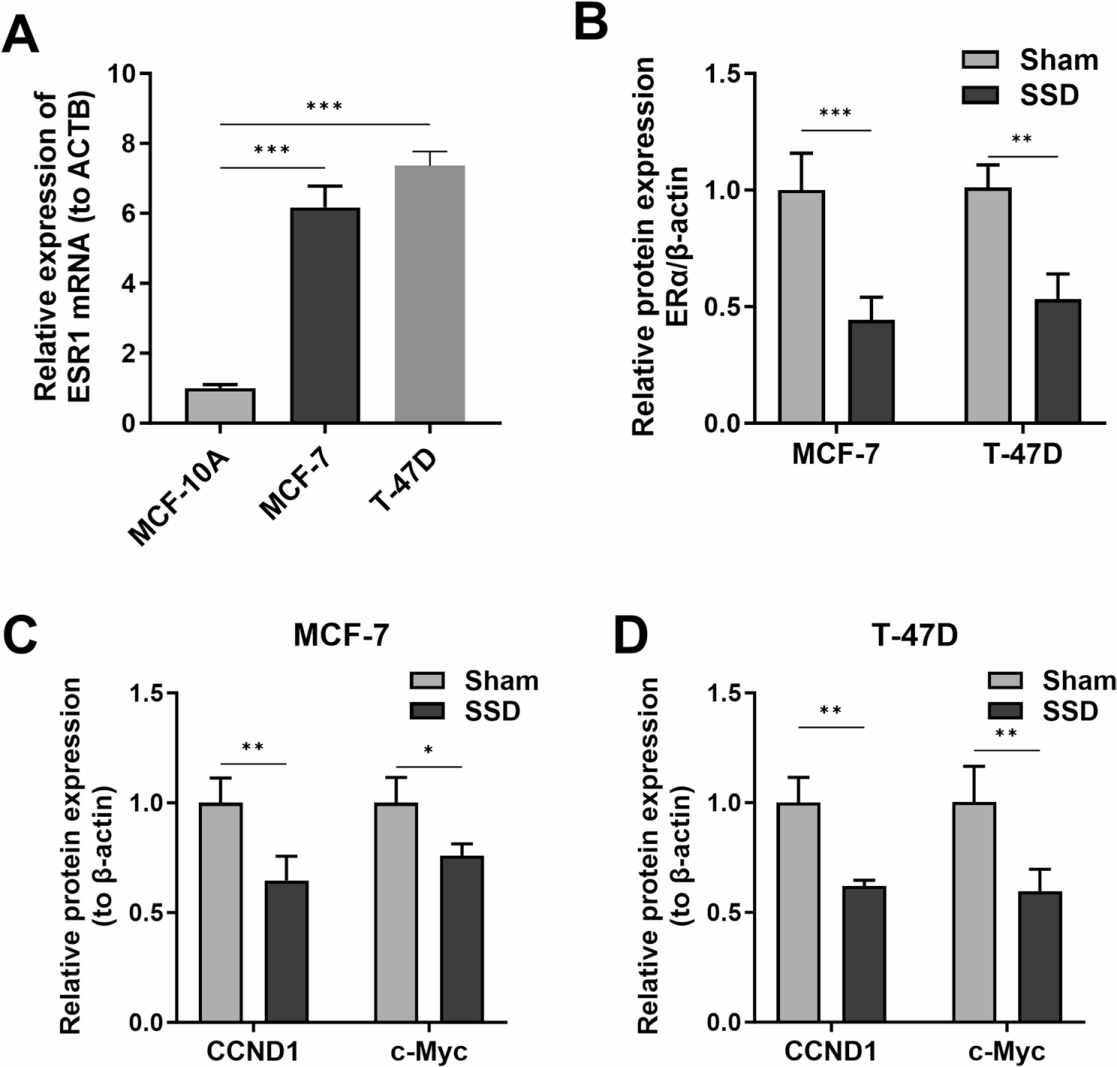
Saikosaponin D inhibited breast cancer may through ESR1/CCND1/c-Myc. (**A**) The expression level of ESR1 mRNA in MCF-7 and T-47D cells by RT-PCR. ***p < 0.001 (One-way ANOVA with the Dunnett post hoc test). (**B**) (**C**) (**D**) The expression level of ERα, CCND1, and c-Myc protein determined by western blotting. ***p < 0.001 (Two-way ANOVA with the Bonferroni post hoc test)

The mRNA expression of ESR1 was increased in MCF-7 and T-47D cells when compared with that in normal MCF-10 A cells (*p* < 0.001; Fig. 5A). ER-α is encoded by the ESR1 gene. After SSD treatment, ERα level was found to be decreased (*p* < 0.01; Fig. 5B). ER-regulated genes, such as c-Myc and CCND1, play a critical role in the proliferation and apoptosis of cells. Therefore, we detected the protein levels of CCND1 and c-Myc in MCF-7 and T-47D cells under SSD treatment. The results showed that SSD can decrease the protein levels in MCF-7 (*p* < 0.05; Fig. 5C) and T-47D cells (*p* < 0.01; Fig. 5D) (Figure S2).

### ESR1 overexpression reversed the effects of SSD

**Fig. 6:**
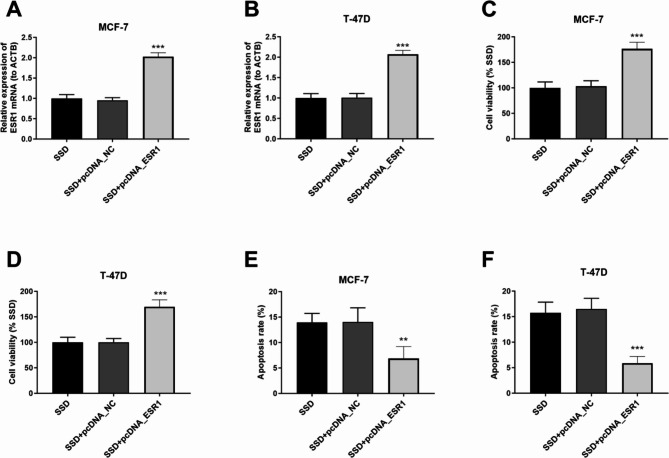
Effects of ESR1 overexpression in Saikosaponin D-treated breast cancer cells. (**A**) (**B**) The expression level of ESR1 mRNA in MCF-7 and T-47D cells by RT-PCR. (**C**) (**D**) Cell viability analysis. (**E**) (**F**) Apoptosis level analysis expressed as percentage of apoptotic nuclei per total nuclei. ***p < 0.001

To test whether the effects of SSD on breast cancer cells could be reversed by ESR1 overexpression, cells were transected with ESR1 pcDNA (*p* < 0.001; Fig. 6A and B). Analysis of cell viability showed that ESR1 overexpression increased the cell viability even under SSD treatment (*p* < 0.001; Fig. 6C and D). On the contrary, overexpression of ESR1 in breast cancer cells reversed the beneficial effects of SSD on cell apoptosis, as cell apoptosis was significantly reduced upon ESR1 overexpression (*p* < 0.01; Fig. 6E and F).

## Discussion

Current anticancer drugs are successful in treating cancer, but may produce negative side effects such as physiological and biochemical changes, exhaustion, and hair loss [22]. By modulating a wide range of biological functions, natural products have been shown to play a key role in preventing and inhibiting cancer. For instance, concanavalin A and silibinin are found to inhibit gatric cancer via attenuation of the JAK/STAT3 signaling pathway based on molecular docking analysis [23]. SSDs have been shown to exhibit anticancer effects by inhibiting initiation, promotion, and progression stages [12]. Our in vitro study showed that SSD could regulate G1/S and G2/M transformation in the cell cycle, inhibit cell proliferation and promote apoptosis in luminal A breast cancer cells. Through network pharmacology and molecular docking simulations, we found that these effects of SSD may be accomplished by targeting ESR1, and subsequently CCND1 and c-Myc.

ER + breast cancer, also known as Luminal A breast cancer, involves hypo-methylated ESR1 or increased ESR1 expression and increased protein levels. We also found increased ESR1 mRNA expression in ER + breast cancer MCF-7 and T-47D cell lines. ERα, encoded by ESR1, is a transcription factor that regulates the expression of genes involved in the cell cycle, proliferation, and apoptosis [24]. Activation of ERα permits the expression of factors such as MYC and CCND1, which have oncogenic potential and increase the risk of cancer cell proliferation and DNA damage in response to estrogen [24]. It has been reported that CCND1 amplification reduced patient’s benefit from tamoxifen, or even aromatase inhibitors, in metastatic breast cancer [25]. We found that SSD can decrease CCND1 level, which highlighted the potential of SSD in the treatment of ER + breast cancer. Overexpression of c-Myc and CCND1 can promote cell cycle progression and oncogenesis. This study revealed the ability of SSD to block the cell cycle at G1.

## Conclusion

In conclusion, the results presented here suggest that SSD can affect the proliferation, cell cycle distribution, and apoptosis of luminal A breast cancer cells. Further, our data suggest that SSD may exert its anti-breast cancer effects via ESR1 and the downstream targets (CCND1 and c-Myc). Together, our study provides in vitro evidence for the role and mechanism of SSD in Luminal A breast cancer. Our findings highlight the anticancer potential of SSD, emphasising its ability to selectively target luminal A breast cancer cells.

## Electronic supplementary material

Below is the link to the electronic supplementary material.

Supplementary Material 1; Figure S1. (A) The OD values of MCF-10A under 9 μM Saikosaponin D. (B) KEGG pathway analysis of the potential targets.

Supplementary Material 2

## Data Availability

The datasets used and/or analysed during the current study are available from the corresponding author on reasonable request.
