# Supplementary figures and images for: The potential mechanism of Saikosaponin D against luminal A breast cancer based on bioinformatical analysis, molecular docking and in vitro studies
Source: Hereditas. 2025 Jul 24;162:140. doi: 10.1186/s41065-025-00510-8 (PMC12291396; doi:10.1186/s41065-025-00510-8)

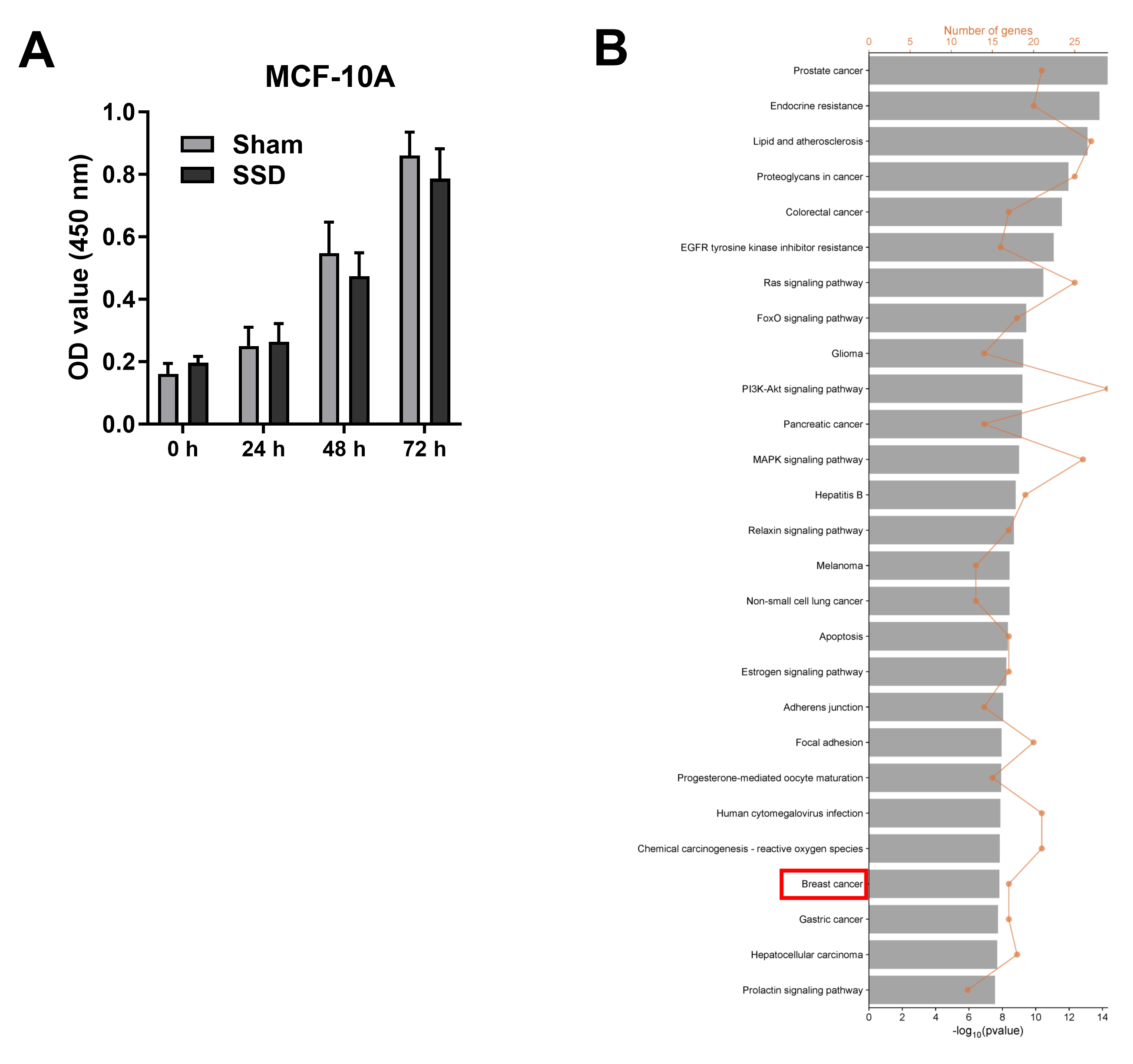

Supplement: Supplementary file 1 — Supplementary Material 1; Figure S1. (A) The OD values of MCF-10A under 9 μM Saikosaponin D. (B) KEGG pathway analysis of the potential targets. [file 41065_2025_510_MOESM1_ESM.tif]

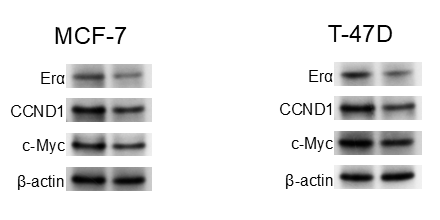

Supplement: Supplementary file 2 — Supplementary Material 2 [file 41065_2025_510_MOESM2_ESM.tif]
